# Parkinson’s Disease Prediction: An Attention-Based Multimodal Fusion Framework Using Handwriting and Clinical Data

**DOI:** 10.3390/diagnostics15010004

**Published:** 2024-12-24

**Authors:** Sabrina Benredjem, Tahar Mekhaznia, Abdulghafor Rawad, Sherzod Turaev, Akram Bennour, Bourmatte Sofiane, Abdulaziz Aborujilah, Mohamed Al Sarem

**Affiliations:** 1 Laboratory of Mathematics, Informatics and Systems (LAMIS), Echahid Cheikh Larbi Tebessi University, Tebessa 12002, Algeria; sabrina.benredjem@univ-tebessa.dz (S.B.); tahar.mekhaznia@univ-tebessa.dz (T.M.); 2 Faculty of Computer Studies (FCS), Arab Open University–Oman, P.O. Box 1596, Muscat 130, Oman; rawad.a@aou.edu.om; 3 Department of Computer Science and Software Engineering, College of Information Technology, United Arab Emirates University, Al Ain 15551, United Arab Emirates; 4 Independent Researcher, Ain Mlila 04002, Algeria; 5 Department of Management Information System, College of Commerce & Business Administration, Dhofar University, Salalah 211, Oman; aaborujilah@du.edu.om; 6 Department of Information Technology, Aylol University College, Yarim 547, Yemen; mohsarem@gmail.com

**Keywords:** Parkinson’s disease, multimodal fusion, attention mechanism, features fusion, artificial neural network

## Abstract

Background: Neurodegenerative diseases (NGD) encompass a range of progressive neurological conditions, such as Alzheimer’s disease (AD) and Parkinson’s disease (PD), characterised by the gradual deterioration of neuronal structure and function. This degeneration manifests as cognitive decline, movement impairment, and dementia. Our focus in this investigation is on PD, a neurodegenerative disorder characterized by the loss of dopamine-producing neurons in the brain, leading to motor disturbances. Early detection of PD is paramount for enhancing quality of life through timely intervention and tailored treatment. However, the subtle nature of initial symptoms, like slow movements, tremors, muscle rigidity, and psychological changes, often reduce daily task performance and complicate early diagnosis. Method: To assist medical professionals in timely diagnosis of PD, we introduce a cutting-edge Multimodal Diagnosis framework (PMMD). Based on deep learning techniques, the PMMD framework integrates imaging, handwriting, drawing, and clinical data to accurately detect PD. Notably, it incorporates cross-modal attention, a methodology previously unexplored within the area, which facilitates the modelling of interactions between different data modalities. Results: The proposed method exhibited an accuracy of 96% on the independent tests set. Comparative analysis against state-of-the-art models, along with an in-depth exploration of attention mechanisms, highlights the efficacy of PMMD in PD classification. Conclusions: The obtained results highlight exciting new prospects for the use of handwriting as a biomarker, along with other information, for optimal model performance. PMMD’s success in integrating diverse data sources through cross-modal attention underscores its potential as a robust diagnostic decision support tool for accurately diagnosing PD.

## 1. Introduction

PD is viewed as a significant neurodegenerative disorder, affecting approximately one million people in the United States and approximately 10 million worldwide [1]. It is characterized by a loss of dopamine-producing neurons, an element of the brain which is crucial for movement control. The root cause of PD remains elusive, though a combination of genetic and environmental factors is likely involved [2]. Despite tireless research efforts and significant advances in clinical management, accurate diagnosis of PD remains a challenge; less than 50% of patients receive a precise clinical diagnosis. It is worth noting that the disease’s pathological processes initiate years before the appearance of observable symptoms, making early detection a daunting task.

During the preclinical stages of PD, subtle alterations begin to unfold, marking a transition from normal functioning to the prodromal state that characterizes the prodromal phase of PD. This stage can persist for years, if not decades, before the clinical onset of PD [3].

Given the current absence of definitive cures for PD, strategies predominantly focus on delaying disease progression. Consequently, the early identification of prodromal PD becomes paramount, serving as a pivotal gateway to initiate preventive interventions before irreversible neurological damage occurs. The early detection process can be significantly enhanced by computer-based tools that can aid in the early detection of PD and prediction of its progression. Deterioration in handwriting may be one of the earliest indicators of illness onset. Handwriting is a process that requires a broad complement of mental, cognitive skills and a notable capacity for sequential activity. In the initial stages of PD, individuals often experience a noticeable reduction in handwriting size, accompanied by difficulties attributed to stiff muscles. This phenomenon is referred to as micrographia or cramped handwriting. Changes in handwriting behaviour should be vigilantly monitored because they can serve as early indicators of PD onset. Consequently, individuals who exhibit such alterations should promptly seek medical evaluation from healthcare professionals.

Recent studies [4,5,6,7,8,9,10,11] have highlighted the significance of handwriting impairment as a crucial indicator of PD, making it a potential biomarker for early PD identification. This underscores the importance of exploring various features associated with micrographia, which is a successful strategy

Compared to laborious and expensive methods such as neurological scanning (e.g., MRI, SPECT), traditional machine learning (ML) algorithms have shown promising results in classifying PD versus HC [9,12,13,14,15,16], though they often face challenges due to the requirement of handcrafted and structural features. Conversely, Deep Learning (DL) and Convolutional Neural Network (CNN) techniques have garnered attention owing to their ability to automatically extract salient visual features from datasets across different domains [16,17,18,19,20]. DL architectures have been used in recent years for early PD diagnosis using various data sources, such as handwriting samples, speech signals, facial features, and gait patterns, making them a focal point in medical and image processing research [21]. In addition, the emergence of ensemble learning and fusion techniques represents a combination set of feature vectors derived from different deep neural architectures into a unified vector. These fusion methods have demonstrated enhanced efficiency and robustness in problem prediction, achieving higher accuracy than single-model approaches.

In this study, we propose a novel Parkinson Multi-Modal Prediction Framework (PMMD). It incorporates a cross-modal attention scheme to integrate Arabic handwriting images, spiral drawing images, and structured clinical data. Our treatment mechanism analyses interactions between these diverse data modalities, thereby improving early detection and diagnosis of PD. However, similar studies used metadata and image features without taking into account the common relationship between the two modalities. We argue that metadata offers extra information leading to good interpretation of image content. In addition, image features enclose information that helps users to understand metadata. The multi-data modalities facilitate each other’s identification of the most relevant features leading to disease classification.

The remainder of this paper is as follows: Section 2 provides an overview of relevant literature in the considered field, while Section 3 provides a comprehensive description of the material used for evaluation of the proposed methodology. Section 4 presents a set of experimental tests and results, followed by a detailed discussion of our findings in Section 5. Finally, Section 6 presents our conclusions. Furthermore, this research not only addresses the underlying causes of PD but also places significant emphasis on early detection and intervention. This aspect has emerged as a focal point in ongoing efforts to combat the disease’s debilitating effects.

## 2. Related Works

In recent years, AI technologies have made great strides in healthcare industries, where deep learning methodologies have become an essential tool for disease diagnosis and prognosis. This section delves into the extensive content of previous research which has used AI and ML/DL techniques in diagnosing PD. The related studies highlight innovative approaches using handwriting samples. To understand such techniques and methodologies, it is crucial to explore the contributions of various research groups that have made significant strides in PD detection using ML/DL techniques.

Pereira et al. [13] proposed a CNN-based model with fine-grained features and a softmax function. The model was trained on samples from multiple datasets leading to an accuracy of 88.19% in PD diagnosis via handwriting analysis. This study highlights the importance of using multiple datasets and advanced models in improving disease diagnosis accuracy.

Khatamino et al. [4] proposed a CNN-based DL system that extracts features from spiral drawings of patients undergoing PD. Experiments achieved an accuracy of more than 88% in recognising PD. The study emphasized the potential of PD diagnosis through handwriting analysis.

Similarly, Rios-Urrego et al. [22] introduced a classification method based on kinematic, geometric, and nonlinear dynamic analyses to differentiate between PD and healthy subjects. By using SVM, KNN, and Adaboost classifiers, experiments achieved an accuracy of 93.1% in diagnosis.

Chakraborty et al. in [23] analyse handwritten patterns like spirals and waves, achieving an overall accuracy of 93.3% using CNNs. Related classifiers demonstrate the efficacy of advanced neural network architectures in PD classification based on handwriting patterns.

Moetesum et al. [24] proposed a multiple networks system for extraction of visual features from diverse graphomotor samples. The system integrates multiple representations and SVM classifiers. It achieves 83% accuracy using only visual information, highlighting the potential of visual-based approaches in PD diagnosis.

Gazda et al. [25] proposed a pretrained neural network approach for PD detection from offline handwriting. They used the PaHaw and NewHandPD Parkinsonism handwriting datasets. Experiments were based on fine tunings in order to bridge the gap between semantically different source and target datasets and facilitate transfer learning (TL). The proposed approach achieved an accuracy of 94.7% from offline handwriting.

Zhao et al. [26] proposed a hybrid system based on CNN and Bidirectional Gated Recurrent Unit (GRU) for analysing the sequential nature of handwriting data. This approach goes beyond static image analysis and considers the temporal dynamics of handwriting movements. Related experiments achieved a test accuracy of 92.91% in identifying PD. The study demonstrates the potential of capturing temporal features for improved diagnosis.

The recognition of these potential gaps underscores the requirement for new concepts and improvement in ML and DL applications for PD diagnosis based on handwritten samples. Despite the enumerated results, the journey from research to clinical application requires several critical issues to be addressed. First, the extensive validation of datasets is crucial to ensure the performance of models across heterogenous populations. It is necessary to consider data from different demographic groups and understand biases that might be present in the training data.

Furthermore, transfer learning techniques significantly enhance the adaptability of models to new and unseen data and improve their generalizability. In addition, imbalanced datasets lead towards more prevalent classes. New concepts are required for managing such challenges [27].

In addition, computational optimization efficiency is also vital for the deployment of models in clinical settings where resources are limited [28]. Hence, other multimodal data sources, such as handwriting, can provide a more comprehensive understanding of PD and enhance model performance and interpretability.

Lastly, a reliance on single-modal data for PD diagnosis may not capture the complexity of the disease, which is inherently multifaceted. So, addressing these gaps strengthens the scientific rigor of the proposed PMMD method and enhances its practical applicability in improving PD diagnosis and patient care. Hence, tackling these challenges allows the development of robust, reliable, and clinically useful tools for an early and accurate diagnosis of PD. Table 1 show a comparative study of the performance of CNN and ViT-based approaches according to the existing literature.

## 3. Proposed Methodology

PD, which is currently incurable, presents opportunities for effective symptom management and disease progression control. Swift diagnosis plays a pivotal role in the management of motor function fluctuations and establishing treatment plans. Previous research has provided significant advances in PD detection, demonstrating significant potential of ML and DL techniques. Relevant works have used diverse analytical methods, such as kinematic analysis and CNNs.

To address these challenges, we propose an approach that leverages advanced analytical methods to improve early PD diagnosis. Our main objective allows clinicians to better understand their patients, leading to early diagnosis of PD. It safeguards human health and reduces medical costs. The second objective consists in a self-diagnosis process based on handwriting samples with the aim of enhancing individual empowerment in health management.

The proposed PMMD capitalizes on this notion. It integrates handwriting analysis as a key modality, alongside clinical data. This approach allows the prediction of subtle motor abnormalities that may precede overt clinical symptoms, thereby enabling proactive intervention and personalized treatment plans.

**Figure 1:**
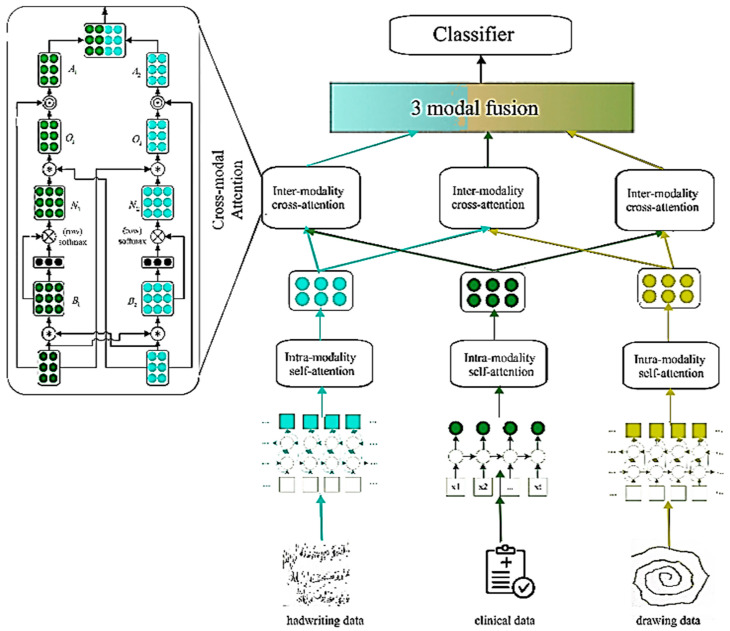
A general schematic of the proposed workflow for the PMMD framework.

In medical diagnostics, clinicians often grapple with diverse data sources, including images and metadata. Integrating this information requires thoughtful fusion techniques, especially concerning dimensional disparities between the imaging data and metadata. The proposed PMMD addresses this challenge by optimizing image and metadata fusion. The PMMD architecture and specific process are illustrated in Figure 1. The PMMD entry considers various heterogeneous data types in input; each is processed along a multi-headed self-attention layer through individual pathways. The process enables modality-specific feature extraction. Subsequently, a cross-attention layer is incorporated in order to enhance decision-making robustness by leveraging cross-modal insights [32]. Then, the fusion of attention computation and cross-modal interactions culminates in a final dense layer that is responsible for prediction.

Hence, the proposed PMMD provides a holistic approach to PD diagnosis, integrating diverse data sources seamlessly to bolster diagnostic accuracy and empower informed healthcare decision-making.

### 3.1. Dataset

The dataset employed consists of a set of handwriting samples created by a group of people who do not all necessarily have mental illness. The acquisition phase was carried out within the Department of Neurology at the Constantine University Hospital Centre (CHU) in Ibn Badis, Algeria [33] from a selected cohort of 150 patients under medical supervision within the psychology, psychotherapy and mental disease departments. Participants of different ages and genders were selected.

**Figure 2:**
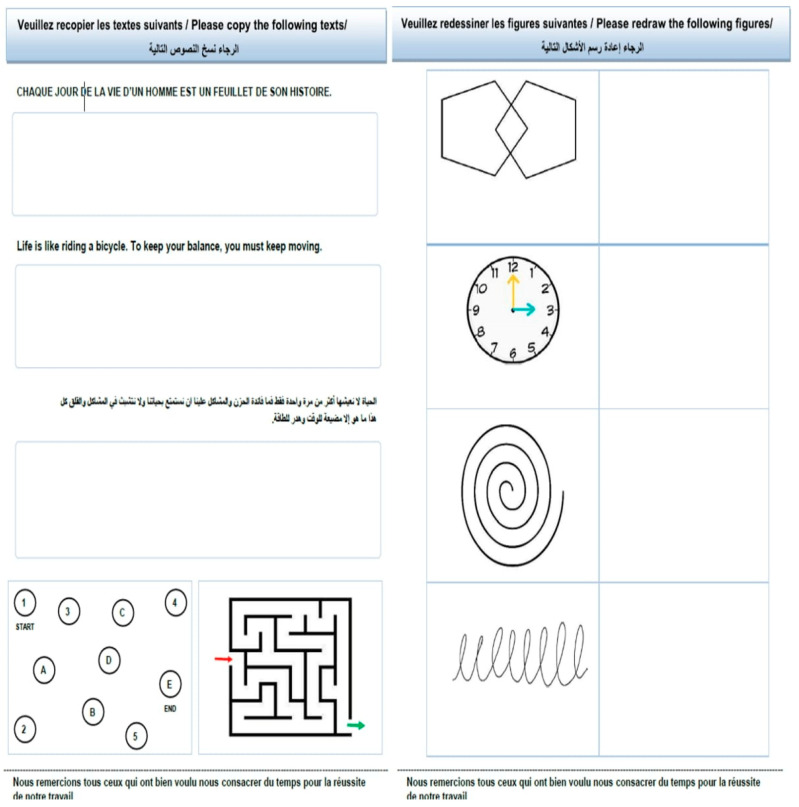
Data collection form.

The samples collected consist of a structured handwriting and drawing task on a printed sheet (Figure 2). This includes intricate handwriting exercises and graphic illustrations to capture several motor and cognitive abilities. To ensure consistent and nuanced data, each task was duplicated to observe subtle performance variations across trials. Participants received clear verbal instructions and were provided with practice opportunities to ensure that they fully understood the tasks and performed at their best. Each participant’s handwritten sheets were digitized using a high-resolution scanner. As a result, data is stored in a well-organized digital format rich with fine details essential for in-depth analysis, preventing any loss of fidelity.

The data collection methodology was designed and standardized to ensure both precision and reliability, providing a strong foundation for subsequent analysis.

**Figure 3:**
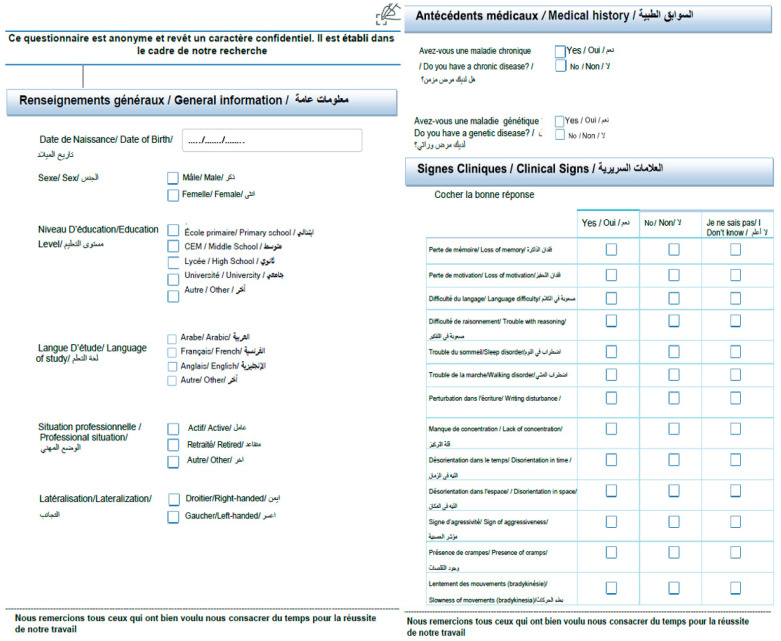
Clinical data form.

The data considered was collected according to a specific protocol including a collection of a comprehensive range of clinical data pertinent to Parkinson’s disease (PD), as illustrated in Figure 3. It consists of a range of personal information such as age at disease onset, educational background, handedness (left- or right-handed), and extensive neuropsychiatric information. Established clinical scales were used to obtain reliable measurements of these variables. This approach allows us to explore potential correlations between handwriting characteristics and the complex clinical profile of PD. It thus provides a unique opportunity to investigate interactions between motor function, cognitive status, and disease progression. This combined dataset enables a comprehensive exploration of PD’s multifaceted impacts, offering profound insights into how motor and cognitive symptoms intertwine, ultimately advancing our understanding of PD’s trajectory.

### 3.2. Data Description

Preprocessing the dataset images improves their quality and enables useful extraction features. The preprocessing mechanism involves various techniques, depending on image characteristics.

#### 3.2.1. Data Preprocessing

**Figure 4:**
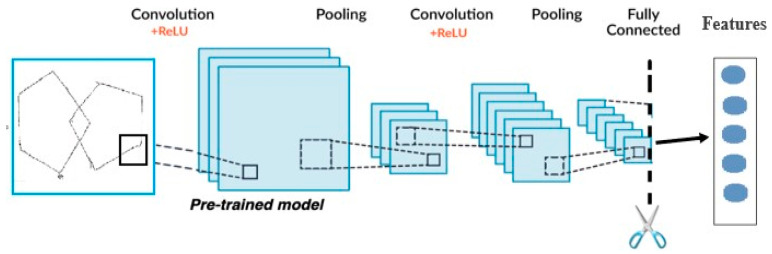
The feature extraction process.

In this stage, we used various preprocessing methods to enhance the quality of writing and drawing data while retaining initial features for subsequent analysis. The preprocessing involves noise removal, binarization, and data augmentation techniques Figure 4. 

**Figure 5:**
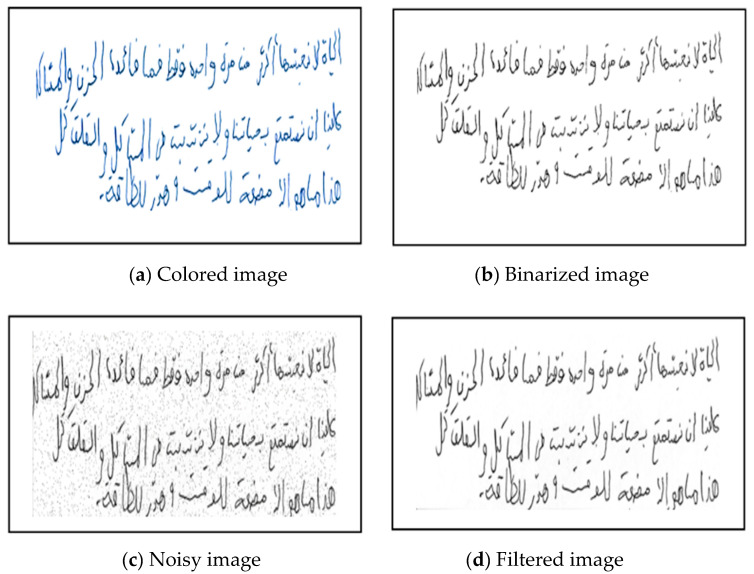
Data preprocessing.

We applied noise removal using a 3 × 3 median filter. We employed OTSU thresholding [34], which reduces image noise and enhances quality. Figure 5 exhibits the preprocessing outcomes applied to a spiral sample. Panel (a) presents the original-coloured sample, and panel (b) displays the binarized version. Panel (c) illustrates a handwritten image with salt and pepper noise, as evidenced in panel (d), which depicts the resultant filtered version without noise. The filtered image (d) is more tuned and refined than the noisy image (c). By removing noise while preserving crucial handwriting details, the filtered image enhances the data quality, leading to more reliable and accurate analysis. This preprocessing step is vital for ensuring the success of the handwriting analysis in PD detection.

**Figure 6:**
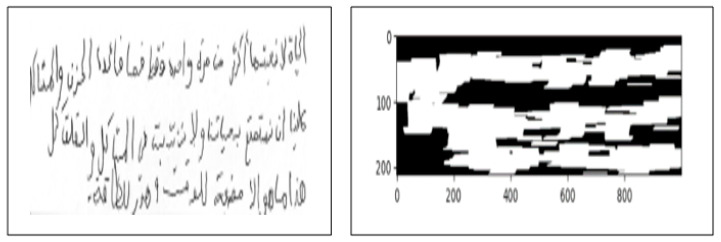
Example of a dilated handwriting image.

Some extracted features seem sensitive to noise, particularly handwriting irregularities and word spacing. This issue is addressed by a two-step process: (a) apply noise removal techniques and then (b) use the dilation process [35], as shown in Figure 6.

In addition, we used data augmentation techniques to enrich the dataset. While we focused on simplicity, future research may explore other techniques such as skew angle correction, thinning, and skeletonization for improved preprocessing. Table 2 summarizes the data augmentation parameters and their corresponding values used in this study.

Overall, these data augmentation parameters were carefully chosen to create a larger and more diverse dataset of handwritten and drawing images while preserving the essential features relevant to PD diagnosis.

#### 3.2.2. Clinical Data Preprocessing

Before inputting the datasets into the machine learning model, an array of preprocessing techniques is systematically applied to ensure optimal performance. Preliminary tasks involve the identification and elimination of outliers, handling missing values via robust imputation methods, standardizing data to mitigate scale discrepancies, and encoding categorical variables for seamless integration into the analytical framework. These steps enhance the model’s efficacy and reliability in discerning patterns indicative of PD within clinical data, including age, sex, and symptomatology.

### 3.3. Features Extraction

The proposed PMMD employs a hybrid feature extraction network specifically designed for multi-modal data analysis. It ingests a combination of images (handwriting and drawings) and clinical data to leverage a unique approach for extracting comprehensive features.

The core component of the feature extraction network is the ability to combine handcrafted features with CNN features. It captures essential details (Pen Pressure Irregularity, Slant Irregularity, Handwritten Lines Irregularity, and Word Spacing) of handwriting and images to provide fine-grained information about the way that a user creates these marks. Concurrently, CNN features are extracted through convolutional and pooling operations.

The feature extraction network seamlessly integrates clinical data to extract features related to patient demographics, medical history, and diagnostic information. This comprehensive approach fosters a holistic understanding of the data, allowing us to consider the physical characteristics of the handwriting/drawings in the patient’s broader medical context.

#### 3.3.1. Spiral Data

Nine statistical features were proposed. They evaluate the Spiral Template (ST) and Drawing Trace (DT) samples and compute their differences. The assessment involved sampling multiple points at corresponding positions in ST and DT images. However, before delving into the discussion of these extracted features, it is imperative to comprehend the concept of the “radius” related to spiral points. It refers to the straight-line distance connecting the centre of a spiral to a specific sampled point. By considering the handwritten trace (HT) and the Exam Template (ET), the radius is evaluated according to the formulae illustrated in Table 3.

#### 3.3.2. Handwriting Data

Within this context, we extracted two different types of features. The first one uses image processing and manipulation functions to process images and extract desired features. The second employs pre-trained CNN networks for extracting features automatically from handwritten documents.

- Feature Extraction Using Image Processing Techniques

In the PD detection field, a sophisticated and comprehensive array of features is extracted from Arabic handwritten images. This delves deeply into intricate nuances of handwriting patterns for identifying potential markers indicative of PD. The key features examined are Pen Pressure Irregularity (PPI), Slant Irregularity (SI), Handwritten Line Irregularity (HLI), and Word Spacing (WS). They offer profound insights through their distinct characteristics:

The PPI feature is designed to assess the uniformity of pressure applied during writing. In the context of PD detection, deviations in pen pressure, characterised by fluctuations or inconsistencies, reveal underlying motor control challenges, potentially serving as a significant diagnostic indicator. By scrutinizing these irregularities, valuable insights are extracted to identify the distinctive handwriting traits associated with PD. The standard deviation (SD) and mean value *x*′ are used to extract the PPI using Equations (1) and (2), respectively.

(1) $$SD=\sqrt{\frac{1}{N-1}*\sum_{i=0}^{n}h\sum_{j=0}^{m}{\left(x-x’\right)}^{2}}$$

(2) $$x’=\frac{\sum_{i=0}^{n}\sum_{j=0}^{m}X(i,j)}{N}$$

where *x*′ denotes the mean value of the pixel intensities of the square *X*, *N* is the number of pixels in a square *X*(*n*,*m*), and *X*(*i*,*j*) is the intensity value of a given pixel.

The SI pertains to deviations in the angle or slope of a handwritten text line. It involves variations in the tilt or slant of individual lines of text within a document. Assessing SI reveals changes in writing angles; it shows motor control challenges, tremors, or muscle stiffness associated with PD. Equation (3) calculates the slant angle of detected lines based on trigonometry.

Angle = arctan2(*y*_2_ − *y*_1_, *x*_2_ − *x*_1_) (3)

The HLI denotes the variation in the alignment, spacing, and curvature of the lines of a handwritten text. This fact encompasses factors such as line spacing consistency, smoothness of line edges, and overall uniformity of the inline shapes. In the PD context, this feature plays a crucial role in detecting patterns and abnormalities in handwritten text, providing valuable insights for diagnostic purposes.

The WS refers to the spacing between successive words in the handwritten text. Within PD detection, diminished spacing between words, a condition known as micrographia, indicates signal motor control difficulties. This fact is viewed as a distinctive trait commonly observed in individuals with PD. Examination of these aspects aids in the recognition of atypical handwriting patterns linked to the condition.

b. Automatic Feature Extraction using Pretrained Deep Neural Networks

In this study, we harnessed the power of pre-trained CNNs, particularly ResNet [15,36], to automate feature extraction from manuscripts. By using transfer learning, we leveraged the extensive knowledge ResNet has acquired from training on the ImageNet: a dataset of over a million images. ResNet’s advanced architecture, featuring skip connections and residual blocks, effectively mitigates the vanishing gradient problem, leading to accelerated learning and enhanced performance.

The proposed Feature Extractor method capitalizes on pre-trained layers for one-time robust feature extraction. It then avoids the need for additional image processing steps. It creates a new enriched dataset based on available features.

ResNet, combined with transfer learning, allows high-quality feature extraction and maximises the model’s utility by training on a vast and varied dataset [9,37]. The structural design of the proposed Transfer Learning model for feature extraction is illustrated in Figure 6.

#### 3.3.3. Clinical Data

Following extensive consultations with pathologists, a selection process was undertaken to identify representative features from traditional medical records that align with the established diagnostic criteria for PD [34]. These records include detailed information about each patient’s medical history, diagnoses, treatments, and outcomes. The extracted features were analysed and correlated with clinical markers recognized in professional practice. This correlation process involved aligning record content with PD symptoms and diagnostic criteria. The process provides an exhaustive clinical profile for each patient, spanning a wide spectrum of domains. It includes crucial demographic details, such as age, gender, ethnicity, family medical history and other essential information related to patient background. Furthermore, it incorporates detailed clinical manifestations, addressing both motor symptoms (e.g., tremors, bradykinesia, rigidity, and postural instability) and non-motor symptoms (e.g., cognitive impairment, mood disorders, sleep disturbances, and autonomic dysfunction) [38].

The collected data is structured according to clinical decision-making (Table 4), providing healthcare professionals with a holistic view of the patient’s condition and supporting the development of personalized treatment plans.

### 3.4. Neural Network Attention

Based on the nature of the inputs used for multimodal fusion, fusion strategies are delineated as feature-level and decision-level fusion. In this study, we selected feature-level fusion; it combines images and meta-information according to their respective handwriting feature (x), drawing (xdrt), and clinical data (xmeta) in the channel dimension using an attention-based multimodal fusion module. It consists of assigning different weights to each feature according to their importance in the diagnostic process. The overall design of the fusion module includes intra-modal self-attention and inter-modal cross-attention.

#### 3.4.1. Intra-Modality Self-Attention

Within each modality, we meet irrelevant information (e.g., background noise in images). To avoid such pitfalls, a multi-head self-attention module is applied to each of the two features. A typical self-attention module consists of several steps: (a) linear projection of the input feature *x* into Query (*Q*), Key (*K*), and Value (*V*) vectors, followed by (b) multiplying the *V* value by the attention weight obtained from the dot-product of *Q* and *K*. The weighted feature *x*′is produced as a result, as shown in Equations (4) and (5).

(4) $$Q=w_{q}x,\;K=w_{k}x,\;V=w_{v}x$$

(5) $${\;\;\;x\;}^{’}=Softmax\left(\frac{{QK}^{T}}{\sqrt{d}}\right)V$$

#### 3.4.2. Intra-Modality Cross-Attention

The purpose of inter-modality is to enhance the model’s interpretation of handwritten data for more robust decision-making. This module incorporates two paths which receive information from each pair of modalities. The first, meta-information, guides the selection of the most relevant information from handwritten features. It is fitted with a cross-attention module with an input vector *Query*, a Value from *wrt* projection, and Key from *meta*. The second reverses this approach by using handwriting features to guide the selection of pertinent information from the metadata. It is fitted with a cross-attention module with an input vector *Query*, a Value from *meta* projection, and Key from *wrt*, as shown in Equations (6) and (7):

(6) $$Q_{1}=w_{q1}x_{wrt},\;K_{1}=w_{k1}x_{meta},\;V_{1}=w_{v1}x_{wrt}\;{x\prime \prime }_{img}=Softmax\left(\frac{{Q_{1}K_{1}}^{T}}{\sqrt{d}}\right)V_{1}$$

(7) $$Q_{2}=w_{q2}x_{meta},\;K_{2}=w_{k2}x_{wrt},\;V_{2}=w_{v2}x_{meta}\;{x\prime \prime }_{meta}=Softmax\left(\frac{{Q_{2}K_{2}}^{T}}{\sqrt{d}}\right)V_{2}$$

### 3.5. Classification and Recognition

The proposed PMMD employs a support vector machine (SVM). An SVM excels in finding the optimal hyperplane in multidimensional space in order to distinguish data points at different classes by using the *rbf* kernel function for classification, i.e., PD and Healthy. This allows the SVM to adjust the nonlinear fitting capacity, leading to potentially superior classification performance compared to other ML methods.

#### Evaluation Metrics

The proposed PMMD diagnosis employs various evaluation metrics to assess its effectiveness, namely accuracy (*ACC*), Sensitivity/Recall (*SN*), Precision (*PR*), and F1-Score (harmonic mean of recall and precision). These metrics evaluate the quality and performance of the employed learning models.

Accuracy determines the percentage of correct predictions produced by the model. A high accuracy score indicates that the model made globally correct predictions. It is illustrated by Equation (8).

(8) $$ACC=\frac{TP+TN}{TP+TN+FP+FN}$$

Precision measures the amount of true positive predictions among all positive predictions performed by the model. A high precision score denotes a positive prediction generated by the model. It is illustrated by Equation (9).

(9) $$PR=\frac{TP}{TP+FP}$$

The recall metric measures the proportion of true positive predictions among all true positive cases of data. A high recall score indicates that the model correctly identifies positive PD cases. It is illustrated by Equation (10).

(10) $$SN=\frac{TP}{TP+FN}$$

The F1 score provides a balance between the harmonic mean of precision and recall. A higher F1 score denotes a better overall performance in terms of identification of positive cases. It is illustrated by Equation (11).

(11) $$F1-score=\frac{2\;*\;PR\;*\;SN}{PR+SN}$$

## 4. Results

### 4.1. Performance of Baseline Model

**Figure 7:**
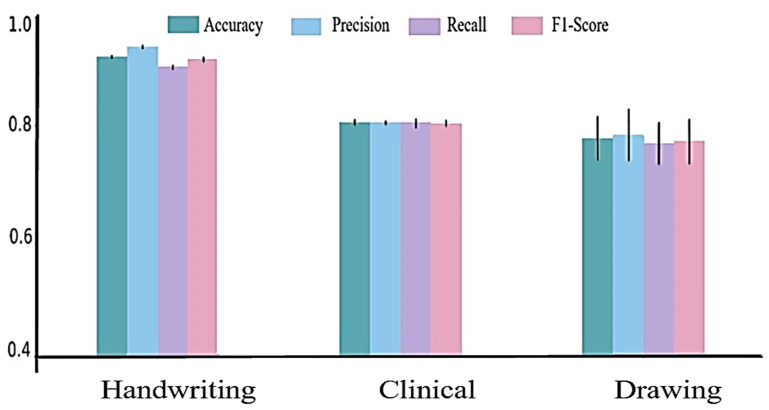
Performance of evaluation metrics.

We first evaluated the performance of a single-modality model as a baseline for further comparisons. In order to illustrate the efficacy and robustness of the proposed multimodal framework, we conducted an assessment by using a single-modality model as a benchmark. The evaluation process uses neural networks and parameter tuning to optimize model performance across all modalities. The data were partitioned into training and testing sets. The reported test accuracies were averaged over five random initializations. The results are presented in Figure 7.

The graph in Figure 7 shows results of evaluation metrics for the best neural network model for the modalities considered (handwriting, clinical, and drawing). It appears that the results of written information perform well in comparison to drawing data, which has the lowest performance of the modalities. For the handwriting images, we used a neural network with three fully connected layers. The test achieved an average accuracy of 92.28%. For clinical data, our system involved a neural network with two fully connected layers; it achieved an accuracy of 80.7%. The sketching image modality achieved an acceptable accuracy of 77.4% in its best unimodality by using a convolutional neural network with three layers.

### 4.2. Performance of the Multimodal Model

To assess the effectiveness of multimodal PD and identify the most informative data combinations, a series of experiments were conducted. They explored the impact of combinations of different modalities (handwriting, clinical data, and drawings) on prediction accuracy. Table 3 delineates the accuracy metrics of the models leveraging diverse modality combinations, with the optimal outcomes highlighted.

As showed in Table 5, the fusion of handwriting data and clinical data emerged as the optimal two-modality combination, exhibiting an accuracy of 93.2%. Furthermore, the inclusion of drawing data alongside handwriting and clinical data in a three-modality fusion resulted in a significant accuracy of 96%, which endorses the multimodal fusion performance. This is due to the significant volume of input data and its diversity which improve the results quality.

In addition, we notice the high performance of experiments using handwriting modalities compared to others (drawing and clinical). This is due to the fact that handwriting samples enclose more specificities and features and, consequently, provide more accurate outcomes.

## 5. Discussion

**Figure 8:**
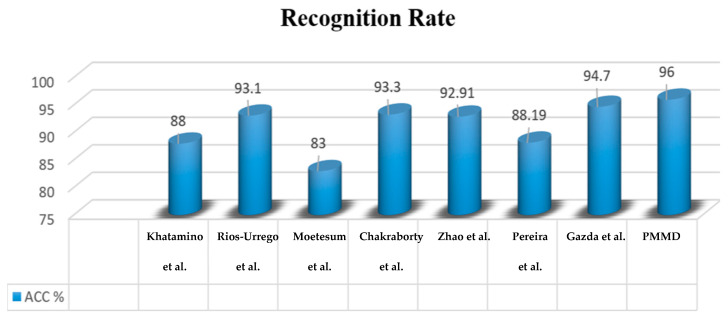
Comparative study vs. similar research [4,13,22,23,24,25,26].

Based on the results shown above, Figure 8 illustrates a comparative study of the proposed model in regard to similar work in the literature. It is apparent that our model outperforms the others in terms of accuracy. It achieved an accuracy of 96%, surpassing Gazda et al. [25] (94.7%), Chakraborty et al. [23] (93.3%), and Rios-Urrego et al. [22] (93.1%).

Previous models demonstrate solid performance, but they lack a degree of precision, underscoring PMMD’s advantage in leveraging both handwriting characteristics and clinical data through an advanced multi-modal fusion strategy.

Our PMMD’s performance is due to the attention mechanism that fine-tunes the weighting of different input features. The attention-based fusion method enhances the model’s sensitivity to subtle PD indicators and addresses the heterogeneity in PD symptom presentation. Also, due to the integration of nuanced patterns from multimodal data sources, our PMMD model built a comprehensive representation of the patient profile, leading to more accurate predictions. This robust, data-driven approach underscores PMMD as a pioneering tool in PD prediction, setting a new benchmark for accuracy and reliability in clinical AI applications.

## 6. Conclusions

In this study, we introduced PMMD, which leverages attention-based deep learning techniques to detect PD using three distinct modalities: handwriting, drawing, and clinical data. Benefitting from multimodal inputs, the proposed model provides a multitask classification and captures inter-modal interactions based on a cross-modal attention mechanism.

In addition, the proposed model considers heterogeneous features by employing attention modules in order to reinforce crucial elements extracted from neural network backbones and cross-modality attention to endorse the relationships between these modalities. This combination yielded an impressive accuracy of 96%, surpassing various similar models. This underscores the importance of attention-based models in clinical settings. As future work, we plan to extend the approach to other types of medical data such as brain imaging (MRI, PET, etc.) by using more diverse datasets.

Overall, the results of this study highlight exciting new prospects for the use of artificial intelligence in medicine.

## Figures and Tables

**Table 1:** Comparative study of related state of art.

Author	Approach	Methodology	Classification Method	Accuracy (%)
[13]	PD identification	Pen-based features-based	ImageNET CIFAR-10 LeNet	91.79
[4]		Handwriting Drawing	CNN-based DL	93.33
[22]		features based on kinematic, geometrical and non-linear dynamic analyses	K-nearest neighbors, support vector machines, and random forest	83.3
[23]		spiral and wave sketches	CNN	93.94
[24]		kinematic and spatio-temporal features	Support Vector Machine	83
[25]		Parkinsonian handwriting dataset (PaHaw) [29] NewHandPD dataset [13]	multiple-fine-tuned CNNs	94
[27]		sEMG signals	deep transfer learning ML SVM	99
[28]		meander, spiral, voice, and speech-Sakar	crow search algorithm and decision tree (CSADT)	100
[30]	PD with healthy control	Progression Markers Initiative (PPMI) database [31]	vector machine (SVM), random forests (RF), K-nearest neighbor (KNN) and artificial neural network (ANN)	96.88

**Table 2:** Data augmentation parameters.

Augmentation Parameters	Setting
Horizontal Flip	True
Vertical Flip	True
Width Shift Range	0.1
Height Shift Range	0.1
Brightness Range	(0.5, 1.5)
Rotation Range	360
Zoom Range	0.2

**Table 3:** Features description.

**Feature**	**Meaning**	Formula
×1	Root Mean Square (RMS) of difference between handwritten trace (HT) and (ET) radius	$\mathrm{RMS}=\sqrt{\frac{1}{n}\;\sum_{i=1}^{n}{\left(r_{HT}^{i}-r_{ET}^{i}\right)}^{2}}$
×2	maximum difference between the HT and ET radius	$\Delta_{max}=\arg max\{|r_{HT}^{i}-r_{ET}^{i}|\}$
×3	minimum difference between the HT and ET radius	$\Delta_{min}=\arg max\{|r_{HT}^{i}-r_{ET}^{i}|\}$
×4	standard deviation of the differences between the HT and ET radius	
×5	Mean Relative Tremor (MRT)	$\mathrm{MRT}=\frac{1}{n-d}\sum_{i=d}^{n}\left(r_{ET}^{i}-r_{ET}^{i-d+1}\right)$

**Table 4:** Clinical variable descriptions.

Feature	Description
Age	The patient’s age at the time of assessment is a crucial factor in PD prevalence.
Gender	Identification of the patient’s sex, considering potential differences in disease presentation.
Education Level	The level of formal education acquired by the patient may have implications for disease risk.
Postural Instability	Impaired balance and coordination often lead to falls and injuries in the advanced stages.
Fatigue/daytime somnolence	Pronounced tiredness, lack of energy, and reduced physical and mental stamina.
Olfactory loss	The reduction or loss of the sense of smell often is one of the earliest signs of PD. Olfactory loss can precede motor symptoms by several years.
Constipation	This can significantly impact patient comfort and quality of life. It is often related to autonomic dysfunction.
Sleep Disorders	Various disruptions in sleep patterns, including insomnia, restless legs, and REM behaviour.
Speech Changes	Alterations in voice quality, such as softening, slurring, or hesitations during speech.
Lift a heavy object	Difficulty or inability to lift a heavy object above one’s head. This functional limitation highlights the decline in strength and coordination associated with PD.

**Table 5:** Performance of different multimodal models for prediction.

Models	Modality	Accuracy
Unimodal	HANDWRITING	92.28%
	DRAWING	77.4%
	CLINICAL	80.7%
Two-Modality	HANDWRITING + DRAWING	90.1%
	HANDWRITING + CLINICAL	93.2%
	DRAWING + CLINICAL	81%
Three-Modality	HANDWRITING + DRAWING + CLINICAL	96%

## Data Availability

The datasets produced and/or analysed in this study can be obtained from the corresponding author upon reasonable request.
